# Entrapment of an EGFR inhibitor into nanostructured lipid carriers (NLC) improves its antitumor activity against human hepatocarcinoma cells

**DOI:** 10.1186/1477-3155-12-21

**Published:** 2014-05-12

**Authors:** Maria Luisa Bondì, Antonina Azzolina, Emanuela Fabiola Craparo, Chiara Botto, Erika Amore, Gaetano Giammona, Melchiorre Cervello

**Affiliations:** 1 Istituto per lo Studio dei Materiali Nanostrutturati, U.O.S. Palermo, Consiglio Nazionale delle Ricerche, Via Ugo la Malfa 153, Palermo 90146, Italy; 2 Istituto di Biomedicina e Immunologia Molecolare “Alberto Monroy”, Consiglio Nazionale delle Ricerche, Via Ugo la Malfa 153 Palermo 90146, Italy; 3 Lab. of Biocompatible Polymers, Dipartimento di Scienze e Tecnologie Biologiche Chimiche e Farmaceutiche (STEBICEF), via Archirafi 32, Palermo 90123, Italy

**Keywords:** Nanostructured lipid carriers, Tyrphostin AG-1478, Drug release, Hepatocellular carcinoma, EGFR inhibitor

## Abstract

**Background:**

In hepatocellular carcinoma (HCC), different signaling pathways are de-regulated, and among them, the expression of the epidermal growth factor receptor (EGFR). Tyrphostin AG-1478 is a lipophilic low molecular weight inhibitor of EGFR, preferentially acting on liver tumor cells. In order to overcome its poor drug solubility and thus improving its anticancer activity, it was entrapped into nanostructured lipid carriers (NLC) by using safe ingredients for parenteral delivery.

**Results:**

Nanostructured lipid carriers (NLC) carrying tyrphostin AG-1478 were prepared by using the nanoprecipitation method and different matrix compositions. The best system in terms of mean size, PDI, zeta potential, drug loading and release profile was chosen to evaluate the anti-proliferative effect of drug-loaded NLC versus free drug on human hepatocellular carcinoma HA22T/VGH cells.

**Conclusions:**

Thanks to the entrapment into NLC systems, tyrphostin AG-1478 shows an enhanced *in vitro* anti-tumor activity compared to free drug. These finding raises hope of future drug delivery strategy of tyrphostin AG-1478 -loaded NLC targeted to the liver for the HCC treatment.

## Background

Hepatocellular carcinoma (HCC) represents the fifth most common cancer worldwide and third leading cause of cancer-related mortality globally, maintaining a dismal prognosis since intermediate and advanced stages still account for a large percentage of cases [1]. Therapeutic options in advanced stage have been quite limited so far, until the discovery of new therapeutic agents that target the molecular pathways involved in hepatocarcinogenesis [1].

Epidermal growth factor receptor (EGFR) is expressed at high levels in a variety of solid tumors. In HCC, the overexpression of this receptor has been associated with late-stage disease, increased cell proliferation, and degree of tumor differentiation [2-4]. In addition, activation of EGFR pathway is a prognostic predictor of survival in patients with HCC [5]. Therefore, EGFR represents a good potential molecular target for biologic therapy of HCC.

Tyrphostins are protein tyrosine kinase inhibitors. Among them, the tyrphostin AG-1478, 4-(3-chloroanilino)-6,7-dimethoxyquinazoline, a competitive inhibitor of the ATP binding site in the kinase domain of EGFR, inhibits proliferation and induces death of liver tumor cells through EGF receptor-dependent and independent mechanisms [6,7]. Previous studies also revealed that tyrphostin AG-1478 has no cytotoxic effects per se against normal hepatocytes, while it prevents proliferation and induces apoptosis in human HCC cells [6]. Moreover, it enhances the sensitivity to cytotoxic drugs like cisplatin and doxorubicin. Therefore, tyrphostin AG-1478 could be a potential therapeutic drug for the treatment of HCC. However, it has not been proposed as a potential antineoplastic drug in HCC yet.

Recently we have successfully realized novel lipid-based drug delivery systems for several lipophilic anticancer compounds by selecting the proper lipid mixture to obtain nanostructured lipid carriers (NLC) and by using the nanoprecipitation method [8-11]. By *in vitro* studies, we have also demonstrated the increased antitumor efficacy of the drug when loaded into NLC compared with free drug.

Thus, in the present study, we describe the preparation of novel tyrphostin AG-1478 -loaded NLC by selecting the suitable matrix composition in order to achieve the chemical-physical characteristics and release profile suitable for parenteral administration of this drug. Moreover, on the best formulation, *in vitro* cell viability assays were carried out to compare the anti-proliferative activity of the drug entrapped into NLC versus free drug on HA22T/VGH cells.

## Results and discussion

In this paper, we describe the preparation of empty and tyrphostin AG-1478 -loaded Nanostructured Lipid Carriers (NLC) and their characterization from the chemical-physical, technological and biological point of view in order to realize a drug delivery system with suitable characteristics for the treatment by parenteral administration.

Tyrphostin AG-1478, a potent and specific inhibitor of EGFR tyrosine kinase, plays a key role in the control of normal cellular growth and abnormal cell proliferation [12]. This molecule is promising for the therapeutic treatment of highly malignant forms of tumors, but it is poorly soluble in aqueous media. Thus, the formulation of this molecule into colloidal nanoparticulate systems, such as NLC, could give many advantages being these particles already proposed for drug administration in cancer therapy [8,10].

In order to obtain a suitable carrier for tyrphostin AG-1478, four NLC formulations were successfully prepared by using the precipitation technique. In particular, a solid un-pegylated lipid (Compritol 888 ATO) or a solid pegylated lipid (Compritol HD5 ATO) were used to obtain the lipid nanoparticles, respectively named NLC-A or NLC-B; while a mixture between a solid lipid (Tripalmitin) with either un-pegylated (Captex 355EP/NF) or pegylated (Acconon CC-6) liquid lipid were used to obtain the lipid nanoparticles, respectively named NLC-C or NLC-D. The choice of different mixtures of solid and/or liquid lipids is based on the consideration that the use of a liquid lipid to prepare NLC systems could give a higher drug loading capacity and a longer term stability during storage than that obtained by using only solid lipids; while the use of a pegylated lipid could give a surface modification of the obtained nanostructures which could improve their pharmacokinetic behaviour by increasing the mean residence time in the bloodstream [11].

In detail, in order to obtain drug-loaded NLC, each chosen lipid or lipid mixture was melted and tyrphostin AG-1478 was added; then to this solution a warm ethanolic solution of Epikuron 200 was added. Preliminary studies were performed in order to ensure the drug stability above the lipid melting points for a time period required to obtain the nanoparticles. No degradation process occurs on the drug at tested conditions (data not shown). To obtain empty NLC samples, the step involving the addition of the drug to the melted lipid was avoided.

Empty or drug-loaded NLC were produced by dispersing the obtained warm organic solution, containing or not the drug, in a cold aqueous solution containing taurocholate sodium salt under mechanical stirring, to allow the lipid solidification.

Finally, each colloidal aqueous NLC dispersion was purified by exhaustive dialysis and freeze-dried. NLC samples were stored at 4 ± 1°C for successive characterization.

Since some physical-chemical and technological properties such as size, surface charge, polydispersity index (PDI) and loading capacity (LC%) are quite critical for biopharmaceutical behavior of NLC, all the obtained empty and drug-loaded samples, after preparation and purification, were characterized in terms of mean particle size and PDI in different aqueous media (bidistilled water, NaCl 0.9 wt% and PBS aqueous saline solutions). Obtained data are reported in Table 1.

**Table 1:** Mean size, PDI in different aqueous media, Loading Capacity (LC%) and Entrapment Efficiency (EE%) values of empty and tyrphostin AG-1478 -loaded NLC

**Sample**	**H**_**2**_**O**		**NaCl 0.9 wt%**		**PBS pH 7.4**			
	**Size (nm)**	**PDI**	**Size (nm)**	**PDI**	**Size (nm)**	**PDI**	**LC (wt%)**	**EE (wt%)**
Empty NLC-A	139.7	0.38	146.2	0.43	122.2	0.59	----	----
Drug-loaded NLC-A	176.2	0.41	207.2	0.90	145.6	0.79	2.8	28.0
Empty NLC-B	62.6	0.46	120.3	0.75	106.6	0.76	----	-----
Drug-loaded NLC-B	80.1	0.49	108.4	0.49	180.1	0.58	1.7	17.0
Empty NLC-C	175.9	0.34	129.2	0.33	117.1	0.41	----	----
Drug-loaded NLC-C	209.7	0.46	203.7	0.22	248.1	0.29	24.0	70.0
Empty NLC-D	185.5	0.29	125.6	0.28	143.8	0.25	----	----
Drug-loaded NLC-D	189.3	0.42	248.1	0.65	181.5	0.52	24.0	70.0

Data indicate that the average diameter of either empty or drug-loaded NLC samples were in the order of nanometer scale in all aqueous media, ranging between 60 and 250 nm.

However, PDI values are too high for NLC-A and NLC-B samples, obtained by using only a solid lipid, especially in isotonic saline solutions. Otherwise, for those systems obtained by using a mixture between solid and liquid lipids, that is NLC-C and NLC-D samples, PDI values are acceptable in all investigated media.

The results indicate that these systems could be injected intravenously, being the mean size values suitable to minimize the uptake from macrophages of Mononuclear Phagocyte System (MPS) [13]. In this way, these particles could circulate in the bloodstream and potentially accumulate in tumor masses as a consequence of the well-known Enhanced Permeability and Retention (EPR) effect [14,15]. In fact, a critical advantage in treating tumors with nanoparticulate systems comes from the unique patho-physiological characteristics of solid tumors: extensive angiogenesis and hence hypervascularization, coupled with poor lymphatic drainage, which allow a facilitate extravasation into the tumor and EPR effect of colloidal systems [14,15].

In Table 1, the LC% (expressed as weight percent ratio between entrapped tyrphostin AG-1478 and the total dried sample weight) and the EE% (expressed as weight percent ratio between entrapped tyrphostin AG-1478 and total amount of tyrphostin AG-1478 used to prepare the nanoparticles) of drug-loaded NLC are also reported. Also in this case, the best values in terms of LC and EE, evaluated by HPLC analysis on each drug-loaded system (as reported in the Materials and Methods section), were obtained when a mixture of solid and liquid lipids was used as matrix composition. In fact, when tripalmitin mixed with either un-pegylated or pegylated liquid lipid are used as matrix composition (named respectively NLC-C and NLC-D samples), a LC of about 24 wt% was obtained (with a EE of 70 wt%); while when un-pegylated or pegylated solid lipid is used as lipid matrix composition (named respectively NLC-A and NLC-B samples), a LC of 1.7 and 2.8 were obtained (with a EE of 17 and 28 wt%, respectively).

These results can be explained considering an increasing effect of the liquid lipid on the drug solubility into the lipid matrix, as other authors have already reported [11].

The zeta potential values were also determined on the obtained samples, and reported in Table 2.

**Table 2:** Zeta potential values in different aqueous media of empty and tyrphostin AG-1478 -loaded NLC

	**ζ-potential (mV) ± S.D.**		
**Sample**	**In H**_**2**_**O**	**In NaCl 0.9 wt%**	**In PBS pH 7.4**
Empty NLC-A	-20.1 ± 5.5	-12.3 ± 4.5	-19.6 ± 3.3
Drug-loaded NLC-A	-30.3 ± 7.7	-13.6 ± 3.7	-20.4 ± 7.7
Empty NLC-B	-18.7 ± 4.6	-10.4 ± 3.6	-15.4 ± 4.2
Drug-loaded NLC-B	-28.5 ± 10.3	-13.2 ± 2.3	-15.1 ± 6.3
Empty NLC-C	-35.7 ± 5.2	-15.9 ± 4.2	-19.7 ± 5.1
Drug-loaded NLC-C	-46.9 ± 8.4	-13.1 ± 2.9	-19.6 ± 5.4
Empty NLC-D	-36.7 ± 5.9	-09.4 ± 2.5	-16.6 ± 3.9
Drug-loaded NLC-D	-38.4 ± 6.5	-11.4 ± 3.5	-16.6 ± 3.7

These values resulted to be high and negative especially in bidistilled water and decreased in isotonic media such as NaCl 0.9 wt% and PBS aqueous solutions probably for the charge shielding effect of solution ions. However, these values assured a potential stability of all the aqueous NLC dispersions. Moreover, a slight increase of NLC surface charge in the presence of tyrphostin AG-1478 compared to empty systems was evidenced, and this result could be explained considering the drug localization probably also onto the nanoparticle surface.

In order to evaluate the storage stability of the obtained systems, each sample (empty or drug-loaded) was lyophilised and stored at 0°C for 3 months in the dark; after this time, mean size, PDI, zeta potential values and LC were evaluated in bidistilled water. Obtained data, reported in Table 3, showed that all empty and tyrphostin AG-1478 -loaded NLC were stable during storage in the tested conditions, being comparable to those of fresh samples.

**Table 3:** **Mean size, PDI and zeta potential in bidistilled water of lyophilised empty and tyrphostin AG-1478 -loaded NLC after storage at 0**°C **for 3 months in the dark**

**Sample**	**Size (nm)**		**PDI**		**ζ-potential (mV)**		**L.C. (wt%)**	
	**Before storage**	**After storage**	**Before storage**	**After storage**	**Before storage**	**After storage**	**Before storage**	**After storage**
Empty NLC-A	139.7	134.9	0.38	0.39	-20.1 ± 5.5	-19.1 ± 4.5	----	----
Drug-loaded NLC-A	176.2	179.5	0.41	0.44	-30.3 ± 7.7	-29.4 ± 7.9	2.8	2.6
Empty NLC-B	62.6	64.4	0.46	0.49	-18.7 ± 4.6	-19.5 ± 4.9	----	----
Drug-loaded NLC-B	80.1	84.4	0.49	0.50	-28.5 ± 10.3	-29.9 ± 16.2	1.7	1.5
Empty NLC-C	175.9	175.9	0.34	0.34	-35.7 ± 5.2	-35.7 ± 5.2	----	----
Drug-loaded NLC-C	209.7	220.5	0.46	0.48	-46.9 ± 8.4	-42.7 ± 7.3	24.0	21.4
Empty NLC-D	185.5	192.5	0.29	0.32	-36.7 ± 5.9	-32.7 ± 4.4	----	----
Drug-loaded NLC-D	189.3	199.3	0.42	0.48	-38.4 ± 6.5	-36.3 ± 4.4	24.0	21.8

Thanks to all their chemical–physical properties and their stability after storage, these systems could be proposed for the administration by all the routes, also intravenously.

**Figure 1:**
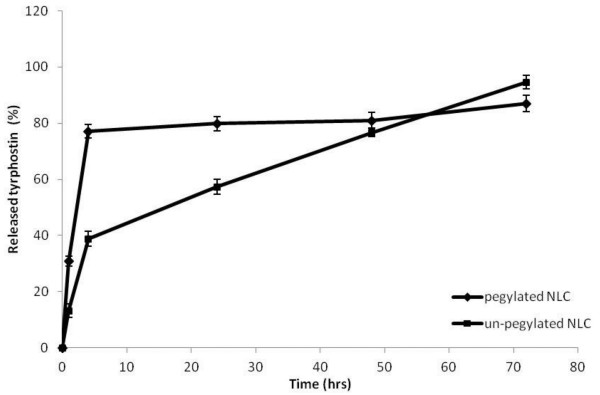
**Release profiles of tyrphostin AG-1478 from NLC-C and NLC-D samples in PBS pH 7.4/ethanol mixture (80:20 v/v) at 37 ± 0.1°C, expressed as percentage wt% of released drug as a function of incubation time.** Each value is the mean of three experiments.

**Figure 2:**
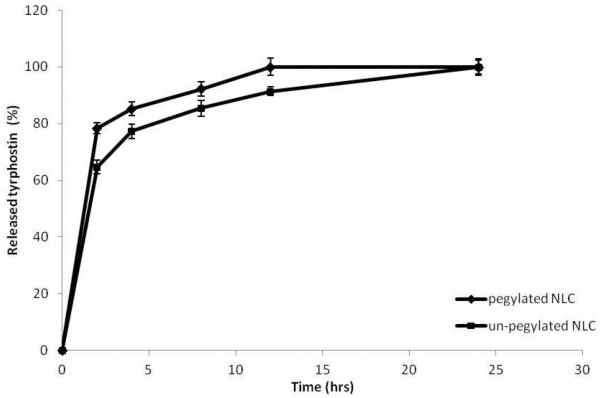
**Drug release profiles of tyrphostin AG-1478 from from NLC-C and NLC-D samples in human plasma at 37 ± 0.1°C, expressed as percentage wt% of released drug as a function of incubation time.** Each value is the mean of three experiments.

All together these results in terms of LC, mean size and zeta potential values indicated that the best NLC to be proposed as drug delivery systems for tyrphostin AG-1478 seem to be those obtained by using the mixture between tripalmitin and the liquid lipid (pegylated or not), that is NLC-D and NLC-C. For this reason, these latter systems were chosen to perform successive characterization in terms of drug release studies and *in vitro* biological assay. In particular, release studies were carried out in different incubating media such as phosphate buffer solution (PBS) at pH 7.4/ethanol mixture (80:20 v/v) or human plasma. The use of this modified dissolution medium (containing ethanol) to test preparation containing poorly aqueous-soluble active substances was in accordance to the European Pharmacopoea [16]. In Figures 1 and 2, the released drug, expressed as weight percent ratio between released drug and the total entrapped drug, is reported as a function of incubation time respectively in PBS/ethanol and in human plasma.

The release trend could be explained considering the high hydrophobic behaviour of the drug that shows a higher affinity for the system obtained by using the un-pegylated liquid lipid mixed with tripalmitin as for lipid matrix composition than for that obtained by using the pegylated lipid. On the other hand, the lower affinity for the pegylated lipid could give a preferential drug deposition in the outsides shell of the NLC during the preparation process, and consequently a burst effect in the release profile of the drug could be evidenced.

Therefore, a modified release of tyrphostin AG-1478 from the un-pegylated systems (NLC-C sample) can be seen in the graphic, being the amount of released tyrphostin AG-1478 about the 90 wt% of the total entrapped amount after 72 hrs incubation.

It was also evaluated that the amount of un-released tyrphostin AG-1478 was still inside NLC sample in the intact form at every incubation time (data not shown). This result supports the great potential of these nanostructures as drug delivery systems for systemic administration of drugs with low solubility and/or instability in aqueous media.

The release profile of tyrphostin AG-1478 was also investigated in human plasma, and obtained data are reported in Figure 2.

Compared to the drug release profiles obtained in PBS at pH 7.4, a faster drug release is evidenced from either the pegylated or the un-pegylated systems in human plasma, probably due to the different composition of the medium, such as the presence of proteins and enzymes in the medium. However, also in this case, the un-pegylated system released the drug slower than the pegylated one. This fact also in this case could be explained considering a higher affinity of the drug for the system obtained by using the un-pegylated liquid lipid mixed with tripalmitin (sample NLC-C).

Showing the NLC-C system the best surface charge and dimensional characteristics useful for parenteral administration and considering their capability to give a controlled release of tyrphostin AG-1478, a further *in vitro* biological characterization was carried out on this system.

**Figure 3:**
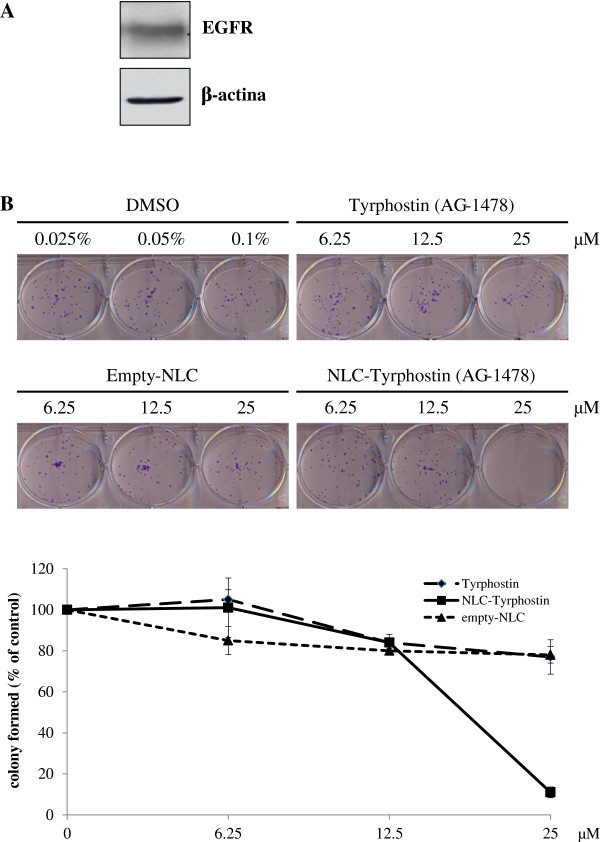
**A) Expression of EGFR in human hepatocellular carcinoma HA22T/VGH cells. B) Effects of free tyrphostin AG-1478, empty-NLC and tyrphostin AG-1478-loaded NLC on the ability of human hepatocellular carcinoma cell line HA22T/VGH to form colony.** Cells were plated overnight and exposed to the indicated concentration of solvent (DMSO), free tyrphostin, empty-NLC and tyrphostin-loaded NLC for 24 hours followed by growth in fresh culture media for 8 days, as described in Materials and Methods. Surviving colonies were stained (upper panel) and counted (lower panel). Data are expressed as a percentage of colony in untreated cells and are the mean ± SD of two determinations.

In particular, the levels of EGFR protein expressed by the human hepatocellular carcinoma (HCC) cell line HA22T/VGH were evaluated by Western blot analysis. As shown in Figure 3A, the cell line intensely expressed the receptor.

Based on this result, HA22T/VGH cells were used for testing the inhibitory effects of tyrphostin AG-1478 free and loaded into the NLC on cell growth. In this regard, the clonogenic assay is currently considered the “gold standard” assay for the assessment of the ability of drugs in killing tumor cells *in vitro* experiments. In fact, this assay is a reliable method to measure the reproductive survival of tumor cells capable of clonal expansion.

In this regard, to evaluate the effect of tyrphostin AG-1478 free and loaded into NLC on the ability to form colonies, HA22T/VGH cells were subjected to clonogenic assay. As shown in Figure 3B, the ability of HA22T/VGH cells to form colonies was slightly inhibited after treatment with free-tyrphostin AG-1478 up to a concentration of 25 μM, whereas, the delivery of tyrphostin AG-1478 from the NLC inhibited colonies formation to approximately 90% at 25 μM, therefore potentiating the activity of tyrphostin AG-1478 to inhibit HCC cell growth.

The results show that tyrphostin AG-1478 -loaded NLC maintain antitumor activity, demonstrating that the entrapment of tyrphostin AG-1478 into NLC does not cause an activity reduction of the drug, but even reduces cell colony survival much more than free drug.

Therefore, the results demonstrate an improved therapeutic efficacy of tyrphostin AG-1478 -loaded NLC compared to the free drug and suggest that lipid nanoparticles could have a great potential as tyrphostin AG-1478 targeted delivery systems.

Finally, considering that solid tumors present much more favorable conditions for preferential accumulation of colloidal sized drug delivery systems such as NLC, these systems can be useful for application in cancer therapy.

## Conclusion

In this paper, the realization of NLC with suitable characteristics for parenteral delivery of tyrphostin AG-1478 in the treatment of cancer was described. In particular, by using the precipitation technique, different lipid compositions were used to obtain the best NLC system in terms of drug loading, mean particle size, and zeta potential values. The amount of tyrphostin AG-1478 entrapped into NLC resulted to be higher into those systems obtained by using a mixture of solid and liquid lipids compared to those obtained by using only the solid lipid. Moreover, the un-pegylated nanoparticles released the tyrphostin AG-1478 slower than the pegylated systems and the amount of unreleased drug was still inside of each nanoparticulate sample. This result supports the great potential of these nanostructures as drug delivery systems with a dispersing and protecting action on drugs, in aqueous media.

Finally, *in vitro* biological characterization was carried out and the HA22T/VGH cells were used for testing the inhibitory effects of tyrphostin AG-1478 free and loaded into the NLC on cell growth. The results show that after treatment with free-tyrphostin AG-1478 up to a concentration of 25 μM the ability of HA22T/VGH cells to form colonies was slightly inhibited, whereas, the delivery of tyrphostin AG-1478 from the NLC inhibited colonies formation to approximately 90% at 25 μM, therefore potentiating the activity of tyrphostin AG-1478 to inhibit HCC cell growth. In conclusion, tyrphostin AG-1478 -loaded NLC maintain antitumor activity, demonstrating that drug activity is not reduced in the presence of the nanoparticle carrier. Moreover, the results demonstrate an improved therapeutic efficacy of tyrphostin AG-1478 -loaded NLC compared to the free drug and suggest that solid lipid nanoparticles could have a great potential as tyrphostin AG-1478 targeted delivery systems for application in cancer therapy.

## Experimental

### Materials and methods

Tyrphostin AG-1478 was purchased from LC Laboratories (PKC Pharmaceuticals, Inc. USA). Tripalmitin (glyceryl tripalmitate) and acetonitrile for HPLC were purchased from Fluka (Milan, Italy). Compritol 888 ATO (mixture of approximately mono-, di- and tri-glycerides of behenic acid at 15, 35 and 50 wt%) and Compritol HD5 ATO (behenoyl polyoxyl-8 glycerides) were gift samples from Gattefossè (France). Captex 355 EP/NF (glyceryl tricaprylate/caprate medium chain triglycerides) and Acconon CC-6 (polyoxyethylene caprylic/capric glycerides) were gift samples from Abitec Corporation (Janesville, USA).

Epikuron 200 (soybean lecithin) was gift sample from Lucas Meyer Company (Germany). Sodium taurocholate was a gift from Prodotti Chimici e Alimentari S.P.A., Basaluzzo (Alessandria, Italy). Water of double distilled quality was obtained from MilliQ Plus systems (Millipore, Germany). The other chemicals, of analytical grade, were obtained from Sigma Aldrich (Milan, Italy).

HPLC (UFLC-Prominence system, Shimadzu Instrument, Kyoto, Japan) was equipped with two pumps LC-20 AD, an UV-visible detector SPD-20 AV, an autosample SIL-20A HT and a column Gemini® C18 Phenomenex (250 mm, 5 μm particle size, 110 Å pores size).

### Preparation of empty and drug-loaded nanostructured lipid carriers (NLC)

Un-pegylated and pegylated NLC, empty or drug-loaded, were prepared by the precipitation method, with appropriate modifications as described previously [9-11]. In particular, a solid lipid (Compritol 888 ATO, 230 mg) or a pegylated lipid (Compritol HD5 ATO, 230 mg) were used to obtain the lipid matrices, respectively named NLC-A or NLC-B; while a mixture between a solid lipid (Tripalmitin, 180 mg) with either un-pegylated (Captex 355EP/NF, 54 mg) or pegylated (Acconon CC-6, 54 mg) liquid lipid were used to obtain the lipid matrices, respectively named NLC-C or NLC-D. As far as of drug-loaded samples preparation is concerned, tyrphostin AG-1478, under mechanical stirring, was added to the melted lipid phase. Preliminary studies were performed, in order to ensure the drug stability above the lipid melting point for a time period required to obtain the nanoparticles. No degradation process occurs on the drug at tested conditions (data not showed). After, an ethanolic solution of Epikuron 200 (48.4 mg/ml) was added and the organic outcome was dispersed into bidistilled water (100 ml) containing sodium taurocholate (177.4 mg) at 2-3°C and stirred by using an Ultraturrax T125 (IKA Labortechnik, Staufen, Germany) at 13,500 rpm for 10 minutes. Finally, the colloidal aqueous dispersion of NLC was purified by exhaustive dialysis in a dialysis tube with 12,000/14,000 Dalton cut-off (Spectra/Por®, California, USA), freeze-dried by a lyophilizer (FreeZone® Freeze Dry System, Labconco Corporation, Missouri, USA) and stored at 4 ± 1°C for successive characterization. Ethanol was completely removed from the aqueous dispersions during dialysis process.

### Particle size determination

The mean diameter and width of distribution (polydispersity index, PDI) of the obtained empty and drug-loaded nanoparticles in aqueous suspension, were determined by Photon Correlation Spectroscopy (PCS) using a Zetasizer Nano ZS (Malvern Instrument, Herrenberg, Germany), which utilizes Non-Invasive Back-Scattering (NIBS) technique. Each sample was appropriately diluted with filtered (0.2 μm) water, NaCl 0.9 wt% and PBS at pH 7.4, and the reading was carried out at 25° ± 1°C and at a 173° angle in respect to the incident beam. When the measurement was carried out in NaCl 0.9 wt%, the instrument setting conditions were: μ = 0.902, RI =1.331; in PBS at pH 7.4, the setting conditions were: μ = 0.980, RI =1.334. In all cases, the temperature of measurements was 25°C ± 1°C. Each suspension was kept in a cuvette and analyzed in triplicate. The deconvolution of the measured correlation curve to an intensity size distribution was accomplished by using a non negative least squares algorithm.

### Zeta potential measurements

The zeta potential values were measured by using principles of laser Doppler velocitometry and phase analysis light scattering (M3-PALS technique).

For this purpose, a Zetasizer Nano ZS Malvern Instrument equipped with a He-Ne laser at a power P = 4.0 mW and with λ = 633 nm was used. Each sample was dispersed in filtered (0.2 μm) bidistilled water, NaCl 0.9 wt% and in PBS at pH 7.4. Instrument setting conditions were equal to those described above for size measurements. Each sample was analyzed in triplicate.

### HPLC analysis and drug loading determination

An adequate HPLC method was developed to reveal tyrphostin AG-1478 and to study its stability in phosphate saline buffer (PBS) at pH 7.4, as well as Loading Capacity (LC%) and drug release profiles from drug-loaded systems. The HPLC analysis was performed at room temperature using the instrument described above. A C_18_ column Gemini (Phenomenex, Hundsfield, UK) packed with 5 μm particles, with dimensions 250 × 4.60 mm i.d., was used for analysis. A mixture of acetonitrile and water (30:70 v/v) containing trifluoroacetic acid (0.1% v/v) with a flow rate of 0.1 ml/min was used as mobile phase.

The peak was measured at a wavelength of 254 nm and quantitatively determined by comparison with a standard curve obtained by using drug solutions in a mixture of acetonitrile:chloroform (1:1 v/v) at known concentrations (t_r_ = 9.50 min). The linearity of the method was studied in the range 5–20 μg/ml.

Loading capacity (LC%) was determined by solving each freeze dried NLC sample (5 mg) in 10 ml of an organic solution of acetonitrile:chloroform (1:1 v/v), filtered with 0.45 μm PTFE filters and analyzed by the HPLC method above described. The results are expressed as actual loading percent (LC%, mg of drug encapsulated per 100 mg of nanoparticles) and encapsulation efficiency (EE%, ratio of actual to theoretical loading). In order to ensure that the drug is not absorbed within the PTFE filters, several tyrphostin AG-1478 organic solutions at known concentrations were filtered and the concentrations values, before and after filtration, were evaluated by HPLC analysis. No significant differences in drug concentrations were evidenced.

### Storage stability

All lyophilised empty and tyrphostin AG-1478 -loaded NLC were stored at 0°C for 3 months in the dark. After this time, samples were dispersed in bidistilled water and characterized in terms of mean size, PDI and zeta potential. Moreover, chemical stability of tyrphostin AG-1478 loaded into the NLC was evaluated by HPLC analysis, as reported above.

### Drug release in PBS at pH 7.4/ethanol

Tyrphostin release was assayed on NLC samples at prefixed time intervals. For this purpose, dispersions of each batch containing 5 mg of each freeze-dried sample in a mixture of 9.6 ml of PBS 0.01 M at pH 7.4 and 2.4 ml of ethanol (80:20 v/v), were prepared and kept at 37 ± 0.1°C under mechanical stirring in a Benchtop 80°C incubator Orbital Shaker model 420 [16]. At scheduled time intervals, solution aliquots (1 ml) were taken out from the outside of the dialysis membrane and replaced with fresh PBS aqueous-ethanolic solution. Release profile was determined by comparing the amount of released tyrphostin AG-1478 as a function of incubation time with the total amount of drug loaded into NLC. Data were corrected taking in account the dilution procedure. A control experiment to determine the release behavior of the free tyrphostin AG-1478 was also performed. A suspension of free tyrphostin AG-1478 in PBS aqueous-ethanolic solution at pH 7.4 was prepared at the same concentration of drug entrapped in the NLC, put into a dialysis tube (MWCO 5,000 Da) and immersed into the proper medium. The amount of tyrphostin AG-1478 was detected as reported above.

### Drug release in human plasma

Tyrphostin release was assayed on NLC samples at prefixed time intervals. For this purpose, dispersions of each batch containing 2.5 mg of each freeze-dried sample in 2 ml of human plasma were prepared and kept at 37 ± 0.1°C under mechanical stirring in a Benchtop 80°C incubator Orbital Shaker model 420. At suitable time intervals, samples were filtered through 0.45 μm nylon filters; then acetonitrile was added and the obtained blend was centrifuged at 4°C and 12,000 rpm for 15 min. Then the supernatant was filtered by 0.45 μm PTFE filters and analyzed by HPLC.

### Western blot analysis

For Western blot analysis whole cellular lysates were obtained using RIPA buffer (Cell Signaling Technologies Inc., Beverly, MA, USA). Protein concentrations of supernatants were determined with the Bio-Rad protein assay kit (Bio-Rad Laboratories SrL, Milan, Italy), and Western blotting were performed as previously described [17], with primary antibodies raised against β-actin (Sigma-Aldrich Srl, Milan, Italy) and EGFR (Cell Signaling Technologies Inc., Beverly, MA, USA).

### Cell culture and clonogenic assay

The human hepatocellular carcinoma HA22T/VGH cell line, a poorly differentiated human hepatocellular carcinoma cell line established from a surgical specimen of hepatocellular carcinoma obtained from a 56-year-old Chinese male was kindly provided by Professor Massimo Levrero (Laboratory of Gene Expression, Fondazione Andrea Cesalpino, University of Rome “La Sapienza”, Rome, Italy) [18]. Cells were cultured in Roswell Park Memorial Institute (RPMI) medium (Sigma, Milan, Italy) supplemented with 10% heat-inactivated fetal calf serum (FCS) (Gibco, Milan, Italy), 2 mM L-glutamine, 1 mM sodium pyruvate, 100 units/ml penicillin and 100 μg/ml streptomycin (all reagents were from Sigma) in a humidified atmosphere at 37°C in 5% CO_2_. Cells having a narrow range of passage number were used for all experiments.

The effect of different inhibitor concentrations on cell growth was assessed using a clonogenic assay. For this analysis, 200 cells were plated onto six-well plates in growth medium and after overnight attachment cells were exposed to various concentrations of solvent (DMSO), free tyrphostin AG-1478, empty NLC and NLC-loaded tyrphostin AG-1478 for 24 hrs. The cells were then washed with medium and allowed to grow for 8 days under inhibitor-free or nanoparticles-free conditions, after which the cell colonies were fixed with 70% ethanol at 4°C for 20 min and stained with crystal violet (0.1% in H_2_O) for 5 min. The plates were rinsed with water, air-dried, photographed and evaluated for colony estimation. Colonies containing more than 50 cells were counted. Relative colony formation was determined by the ratio of the average number of colonies in cells treated with free-tyrphostin AG-1478, empty-NLC and NLC-loaded tyrphostin AG-1478 to the average number of colonies in cells treated with DMSO. All experiments were performed in duplicate and repeated twice.

## Abbreviations

NLC: Nanostructured lipid carriers; HCC: Hepatocellular carcinoma; EGFR: Epidermal growth factor receptor; MPS: Mononuclear phagocyte system; PDI: Polydispersity index; LC: Loading capacity; EPR: Enhanced permeability and retention; EE: Entrapment efficiency; DMSO: Dimethyl sulfoxide; PBS: Phosphate buffer solution.

## Competing interests

The authors declare that they have no competing interests.

## Authors’ contributions

CB and EA are the PhD students who carried out the laboratory work. AA carried out the biological work in laboratory. MC was the supervisor of the biological study and helping to develop the study parameters and design. MLB was the principal, scientific supervisor of the study. She conceived the study, supervised the students in the laboratory, directed the analysis and wrote the manuscript. All authors read and approved the final draft of the manuscript. EFC and GG have revised the final version of manuscript.
