# Correction to: Entrapment of an EGFR inhibitor into nanostructured lipid carriers (NLC) improves its antitumor activity against human hepatocarcinoma cells

**DOI:** 10.1186/s12951-017-0325-y

**Published:** 2018-01-13

**Authors:** Roberto Di Gesù, Maria Luisa Bondì, Antonina Azzolina, Emanuela Fabiola Craparo, Chiara Botto, Erika Amore, Gaetano Giammona, Melchiorre Cervello

**Affiliations:** 1 Polymer Science Lab, Chemistry Dept “G.Ciamician”, Alma Mater Studiorum, University of Bologna, Via Selmi 2, 40126 Bologna, Italy; 2 Istituto per lo Studio dei Materiali Nanostrutturati, U.O.S. Palermo, Consiglio Nazionale delle Ricerche, Via Ugo la Malfa 153, 90146 Palermo, Italy; 3 Istituto di Biomedicina e Immunologia Molecolare Alberto Monroy, Consiglio Nazionale delle Ricerche, Via Ugo la Malfa 153, 90146 Palermo, Italy; 4 Lab. of Biocompatible Polymers, Dipartimento di Scienze e Tecnologie Biologiche Chimiche e Farmaceutiche (STEBICEF), Via Archirafi 32, 90123 Palermo, Italy

## Correction to: Journal of Nanobiotechnology 2014, 12:21 10.1186/1477-3155-12-21

Following publication of our article [1], we became aware that Roberto Di Gesù had been omitted from the list of authors. The corrected author list and authors’ contribution statement appear below. We apologize for any inconvenience this may have caused.

### Corrected author list

Roberto Di Gesù, Maria Luisa Bondì, Antonina Azzolina, Emanuela Fabiola Craparo, Chiara Botto, Erika Amore, Gaetano Giammona, Melchiorre Cervello

### Corrected Authors’ contributions

CB and EA are the Ph.D. students who carried out the laboratory work. AA carried out the biological work in laboratory. MC was the supervisor of the biological study and helping to develop the study parameters and design. RDG performed principal and most important analysis in the chemical and pharmaceutical studies. He produced materials and analyzed data showed in this work. MLB was the principal, scientific supervisor of the study. She conceived the study, supervised the students in the laboratory, directed the analysis and wrote the manuscript. EFC and GG have revised the final version of manuscript. All authors read and approved the final manuscript.

### Competing interests

The authors declare that they have no competing interests.
